# Could LC-NE-Dependent Adjustment of Neural Gain Drive Functional Brain Network Reorganization?

**DOI:** 10.1155/2017/4328015

**Published:** 2017-04-30

**Authors:** Carole Guedj, David Meunier, Martine Meunier, Fadila Hadj-Bouziane

**Affiliations:** ^1^INSERM, U1028, CNRS UMR5292, Lyon Neuroscience Research Center, ImpAct Team, 69000 Lyon, France; ^2^UCBL, 69000 Lyon, France

## Abstract

The locus coeruleus-norepinephrine (LC-NE) system is thought to act at synaptic, cellular, microcircuit, and network levels to facilitate cognitive functions through at least two different processes, not mutually exclusive. Accordingly, as a reset signal, the LC-NE system could trigger brain network reorganizations in response to salient information in the environment and/or adjust the neural gain within its target regions to optimize behavioral responses. Here, we provide evidence of the co-occurrence of these two mechanisms at the whole-brain level, in resting-state conditions following a pharmacological stimulation of the LC-NE system. We propose that these two mechanisms are interdependent such that the LC-NE-dependent adjustment of the neural gain inferred from the clustering coefficient could drive functional brain network reorganizations through coherence in the gamma rhythm. Via the temporal dynamic of gamma-range band-limited power, the release of NE could adjust the neural gain, promoting interactions only within the neuronal populations whose amplitude envelopes are correlated, thus making it possible to reorganize neuronal ensembles, functional networks, and ultimately, behavioral responses. Thus, our proposal offers a unified framework integrating the putative influence of the LC-NE system on both local- and long-range adjustments of brain dynamics underlying behavioral flexibility.

## 1. Introduction

The locus coeruleus-norepinephrine (LC-NE) system is involved in a wide range of cognitive functions including perception, working memory, attention, emotional processes and learning, and memory [1–5]. While its widely distributed projections [6–10] and its involvement in the sleep-wake cycle [11–13] have long confined this neuromodulator to a role in arousal and vigilance [14–17], it is now considered as a system with a more complex role in cognitive functions. The specific topography of the norepinephrine receptors and transporters in the brain represents a key element of this complexity [18]. The impact of norepinephrine signaling on brain activity is the result of a fine balance between excitatory and inhibitory actions via these various receptor types onto the target regions depending on the context [19, 20].

Phasic responses of the LC neurons are triggered by behaviorally relevant stimuli [21–23], novel or salient stimuli [24], and stressors [25, 26] and vary with the level of vigilance [1, 27, 28]. More recent evidence from electrophysiological recordings also suggests an influence of the LC-NE system beyond sensory processing [29] to facilitate behavioral adaptation or flexibility. Based on these properties, several theoretical models have suggested that the LC-NE system orchestrates the transition between different behavioral/cortical states to adjust to the current context [30–35]. For the purpose of this review, we will focus on two influential models suggesting that the LC-NE system facilitates behavioral adaptation by two different, not mutually exclusive, processes: (1) a “reset signal” allowing large-scale brain network reconfiguration to adapt and respond appropriately to the environment [33, 36] and (2) a modulation of neural gain in its target regions that increases the signal-to-noise ratio and tune neural network dynamics to optimize behavioral responses [32, 37, 38]. We will describe these two models and present our recent findings together with new data on NE-dependent modulations of both global and local brain functional connectivity dynamics. In light of these findings, we then propose a NE-dependent mechanism of action at the whole-brain level unifying these two theoretical models. Specifically, we propose that the LC-NE system modulates neural gain locally that in turn drives large-scale brain network reorganizations. We also discuss the functional significance of these local-to-global modulations in brain dynamics driven by the LC-NE system on neural signaling and behavioral flexibility.

## 2. The LC-NE System and Functional Brain Network Reorganization

**Figure 1:**
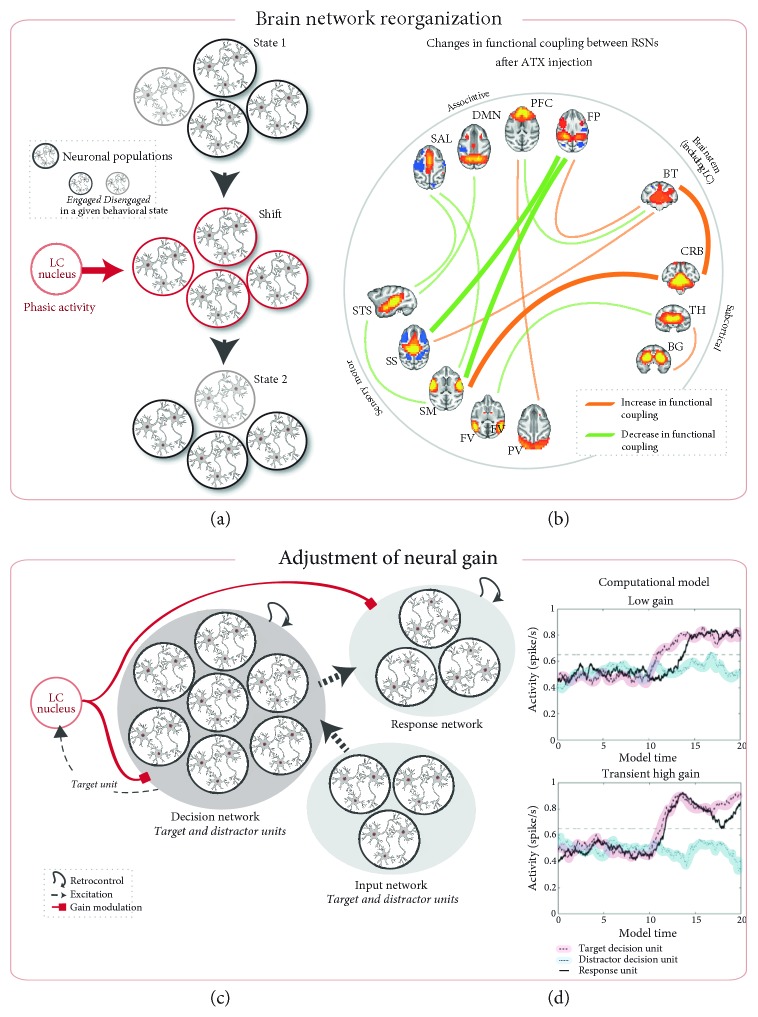
LC-NE system theoretical models ((a) and (b)) brain network reorganizations adapted from Bouret and Sara [33]. (a) A behavioral state is associated with a given functional network with a specific spatiotemporal pattern of neuronal activity. When a stimulus induces a behavioral shift, the LC activation immediately preceding this change modulates the underlying interactions between the neuronal populations via its simultaneous action on several of its target structures, promoting changes within and between functional networks (state 1 → state 2). (b) Overview of the functional coupling changes between 13 resting-state networks (RSNs) following ATX injection (from Guedj et al. [49]). Line thicknesses reflect the correlation strength of the ATX-induced changes. ATX injection modulated the functional coupling of the subcortical network including the LC and decreased the functional coupling between associative and sensory-motor networks. The frontoparietal network, negatively correlated with the brainstem network including the LC region in the saline condition, switched to a positive correlation under ATX ((c) and (d)) adjustment of neural gain. (c) Architecture of the computational model described by Usher et al. [38]. The LC inputs regulate the gain via a multiplier effect on the decision and the response networks. (d) Simulated time courses of activity for the response and decision model units under various neural gain levels (low and high gain). A transient increase of the neural gain induced by a LC phasic response improves the processing of the target stimulus, resulting in faster and sharper increase in response unit activity. Adapted from Usher et al. [38] and Gilzenrat et al. [60]. Circles represent a defined neuronal population. The red circle represents the population of LC neurons. ATX = atomoxetine, BG = basal ganglia, BT = brainstem, CRB = cerebellum, DMN = default-mode network, FP = frontoparietal, FV = foveal visual, PFC = prefrontal cortex, PV = peripheral visual, SAL = salience, SM = somatomotor, SS = somatosensory, STS = superior temporal sulcus, and TH = thalamus.

Bouret and Sara [33] interpreted the NE action from the simplified models of “central pattern generator circuits” of the crustaceans, which have been widely used to explore neuromodulatory mechanisms. These simplified circuits highlighted the capacity of neuromodulators to reorganize or reconfigure neural networks [20, 39]. Bouret and Sara [33] thus suggested that the LC phasic activity plays the role of a “reset signal”, facilitating behavioral transitions. They described an intratask state in which attention is directed toward “expected” and task-relevant stimuli and where behavioral transitions allow the initiation of motor responses required for the current task. For example, in rats performing an odor discrimination task, flashing lights indicating the start of each trial induced an orientating response of the animal toward the port delivering the odor and systematically triggered a phasic LC discharge [40]. Alternatively, the extratask state is described as a state more sensitive to behavioral transitions and attentional reorienting. Bouret and Sara [33] suggest that the reset signal can interrupt ongoing activity in existing functional networks (see also [41]), in order to trigger brain network reorganizations and thus promote the establishment of a new behavior (Figure 1(a)). According to this model, the impact of the LC-NE system would depend on the context and could therefore promote changes within and between any given functional networks in line with the numerous NE-dependent effects observed at the behavioral level.

In line with this hypothesis, Coull et al. [42] demonstrated in a positron emission tomography study conducted in human subjects that during an attentional discrimination task, the administration of clonidine, an *α*_2_ norepinephrine agonist, modulated the efficiency of the connections between the frontal and parietal areas and between the parietal cortex and the thalamus compared to the placebo condition. Another recent functional magnetic resonance imaging (fMRI) study in humans also highlighted a NE-dependent modulation of functional connectivity in the presence of aversive stimuli [43]. Subjects were exposed to aversive stimuli activating and increasing functional connectivity within the salience network, a network including the amygdala, the anterior insula, and the anterior cingulate cortex, and involved in attentional reorientation in response to emotional stimuli [44, 45]. They reported that the administration of a *β*-norepinephrine antagonist reduces the activation and functional connectivity within the salience network in response to aversive stimuli. These studies therefore demonstrate NE-dependent modulation of the functional connectivity within large-scale brain networks.

We recently brought the first empirical evidence that enhancing NE transmission using atomoxetine (ATX), an agent that increases extracellular levels of NE by occupying the presynaptic NE-reuptake transporters [46–48], induces functional brain network reorganizations at rest [49] (Figure 1(b)). In particular, we showed that boosting NE transmission led to (1) a switch in the functional coupling between the brainstem network, that includes the LC nucleus and the frontoparietal attention network, (2) decreased functional connectivity between sensory-motor and associative networks, and (3) decreased correlations within sensory-motor networks. The brainstem network including the LC nucleus, which was negatively correlated with the frontoparietal attention network in the placebo condition, became positively correlated with the latter after ATX administration. Together with the findings of Coull et al. [42] described above, the changes in functional connectivity within and between the frontoparietal attention network and the brainstem nuclei could represent a central feature of the NE action on attentional processes to adjust to the surrounding context [36, 50]. In addition, the decrease in functional connectivity strength between resting-state networks (RSNs) and within sensory-motor networks might reflect a reduction of noise correlation, another feature that could favor stimulus selection [51, 52]. This finding echoes with the electrophysiological studies showing that NE improves perceptual processes within sensory cortices by decreasing the spontaneous neuronal discharges on the one hand and by increasing the evoked responses to the relevant stimuli on the other hand [53–55]. To conclude, according to Bouret and Sara [33], the “reset signal” triggered by the LC phasic discharge would guide the behavior toward the most relevant stimulus of the environment at a given moment. The ability of the LC-NE system to promote behavioral transitions would be achieved through large-scale, behavior-specific reconfigurations of brain networks, depending on the context, thus permitting the expression of a multitude of brain states. Our recent findings provide the first empirical evidence of such NE-dependent large-scale brain network reorganization at rest [49]. Future studies using a whole-brain approach could provide evidence of context-specific brain network reorganizations driven by the LC-NE system.

## 3. The LC-NE System and Neural Gain Adjustment

Another theory suggests a modulation of the neural gain driven by the LC-NE system [32]. Simply explained, neural gain modulations have been suggested to affect neural communication. When neural gain increases, excited neurons become even more active and inhibited neurons become even less active, thus increasing the contrast of the activity pattern in a neuronal circuit [56]. It was suggested that rapid changes in neuronal responsiveness and interactions induced by gain adjustment may trigger dynamic modulations of functional connectivity [57–59]. The model put forward by Aston-Jones and Cohen (*The Adaptive Gain Theory*, [32]) proposed a key role of the norepinephrine system in optimizing behavioral performance, which would involve (1) a regulation of the balance between exploitation and exploration behaviors and (2) an improvement of neural responses to relevant stimuli. Considering the capacity of the LC-NE system to guide transitions between behavioral states in line with Bouret and Sara's proposal, the authors suggest a role in behavioral adjustment, which implies taking part in a fundamental trade-off in their expression: the exploitation of well-known sources of reward against the exploration of the environment looking for other opportunities of higher or more stable value.

In the *Adaptive Gain Theory* largely based on electrophysiological observations in behaving animals, Aston-Jones and Cohen distinguished two distinct modes of activity of the LC neurons: phasic and tonic [1]. In the phasic mode, phasic bursts of LC neurons (i.e., stimulus evoked) are observed in close relationship with goal-directed behaviors. It was proposed that the LC phasic mode would act as an attentional filter for irrelevant stimuli, promoting task-related behaviors. This filter is temporally restricted (to task-related events), but spatially extended given the wide projections of LC neurons [32]. In the tonic mode, spontaneous activity is high while phasic bursts are rare or absent, and behavior is more disorganized. This mode is thought to facilitate shifts of attention and the exploration of alternative opportunities. The LC activity modes would therefore adjust the balance between these two fundamental states: exploitation versus exploration to optimize behavior in a changing environment. According to the *Adaptive Gain Theory* view, the adjustment between exploitation and exploration is associated with a NE-dependent modulation of the neural gain in target areas. Such changes of the neural gain arise in a strategic and time-limited manner and improve locally the signal-to-noise ratio [37, 38, 60, 61] (Figure 1(d)). Usher et al. [38] explored the impact of such changes in neural gain on behavioral performance during an attentional discrimination task. In this task, behavioral responses were modeled in a simplified network in which two alternative representations of the stimulus (target or distractor) compete. The noise in sensory processing related to the perceptual overlap between targets and distractors induces a competition between the neural representations of the two alternatives. In this circuit, LC units received afferent inputs from the decision unit and sent projections back to both decision and response units (Figure 1(c)). In tonic mode, the gain level remained constantly high, inducing a strong competition between neural pools encoding the target (the real “signal”) and the distractor (considered as a “noise”). This condition led to greater variability in reaction times and greater difficulty in discriminating target stimuli. Conversely, in phasic mode, the gain level remained generally low, which leads to a greater resistance to noise. In this state, the presence of a target stimulus elicits a transient phasic discharge that translates into a brief increase of the gain across the network. This transient increase improved the processing efficiency during a specific time window, thus facilitating performance, that is, target discrimination (Figure 1(d)).

A recent human study explored the relationship between neural gain at the whole-brain scale using fMRI and behavioral performance in a learning task [62]. In order to infer the neural gain variations dependent on the norepinephrine system, the authors measured the pupillary diameter. Using a network simulation, they provided mechanistic insights into the link between neural gain, brain-wide neural interactions and topology, and behavioral responses. They explored two brain properties that reflect the functional topology of the brain: the functional connectivity strength (the mean of absolute correlation score between various brain regions) and the clustering coefficient (reflecting the rate of node agglomeration in a network). They observed that a high gain (inferred from a large basal pupillary diameter) was associated with increased functional connectivity strengths and stronger clustering coefficients and vice versa. These results fit with the *Adaptive Gain Theory* and related computational models [37, 38, 60, 61], suggesting that an increase of the neural gain facilitates neural communication.

To summarize, according to the *Adaptive Gain Theory*, cognitive flexibility seems to be associated with variations of the basal (tonic) activity of the LC neurons that would permit a fine regulation of the neuronal activity across the brain via the variety of norepinephrine receptors and their particular topography. This regulation likely involves the interplay between several brain regions such as regions of the frontal cortex [32, 35], together with parietal regions and sensory-motor networks.

## 4. Co-Occurrence of Neural Gain Adjustment and Functional Brain Network Reorganization Induced by a NE Challenge?

In the previous sections, we reviewed theoretical and empirical evidence in favor of a role of the LC-NE system in dynamically modulating both short- and long-range neural dynamics that could permit cortical state adjustment to the changing environment [30, 31]. The next question we ask is how these two mechanisms, namely the large-brain network reorganization and the neural gain adjustment, could interact to facilitate behavioral flexibility. To answer this question, we first attempted to provide evidence of the co-occurrence of these mechanisms at the whole-brain level within the same subject and under the same condition. Providing the evidence of the co-occurrence of a whole-brain network reconfiguration with an adjustment of the neural gain would help better characterize the effect of this neuromodulator. As described above, a recent computational work suggested that an increase in baseline pupil diameter, interpreted as an increase in neural gain induced by a LC-NE activation, was associated with clustered neural interactions [56, 62, 63]. We directly tested this hypothesis by investigating, under a NE challenge, RSN topology in the same dataset as that in Guedj et al. [49] that demonstrated a NE-dependent large-scale brain network reorganization. Here, we used graph theory properties to infer the state of neural gain [62]. Specifically, we characterized the effect of ATX on the quality of information spread (global efficiency and clustering coefficient) and the strength of functional connectivity, at the whole-brain level and within specific RSNs (see Supplementary Material available online at https://doi.org/10.1155/2017/4328015 for the details on the methods).

Briefly, three monkeys participated in the study, as described in Guedj et al. [49]. Resting-state fMRI scans (2 × 2 × 3 mm; TR = 2 s; 400 TRs) were acquired under two conditions: ATX (0.5 mg⁄kg), an inhibitor of NE reuptake, or saline (control condition) injections were administered intramuscularly one hour before the scanning session. Spontaneous slowly fluctuating brain activity (0.01–0.1 Hz) was extracted. Matrices with 471 defined gray matter areas served to construct functional connectivity graphs—one graph per monkey and per run. An area corresponded to a volume of 4 × 4 × 6 mm^3^ (eight voxels) to minimize artifactual correlations between neighboring voxels [64] while retaining a relative fine-grained approximation of the neural gain. Normalized correlations (Fisher r-to-z transformation) between the regional mean time series of each pair of areas were then computed, and a threshold based on the absolute values of their correlation coefficient was applied to retain only the 10% of the highest correlation scores. This density was selected as it was the smallest density that maximizes the number of connected nodes [65] (see Figure S1) while minimizing the number of spurious edges in each area [66]. For each graph, we estimated different metrics: the global efficiency, the clustering coefficient, and the connectivity strength. These metrics were computed for the whole brain and for each of the thirteen “real” networks previously identified with the independent component analysis (ICA) approach [49] (see Supplementary Material for a more detailed description on these metrics). The global efficiency reflects the level of global integration within a network and corresponds to the averaged inverse shortest path length between all pairs of nodes in the network. The clustering coefficient informs us about the “local efficiency” as it reflects the number of connections that exists between the nearest neighbors of a node as a proportion of the maximum number of possible connections [67]. It can be regarded as a measure of information spread in the immediate neighborhood of each node as described above in Eldar et al. [62]. The connectivity strength is defined as the mean of the correlation coefficient between each node and all the other nodes within the network. We then examined the effect of ATX on these three metrics using a linear mixed model, including the pharmacological condition as fixed factor and the subject as random intercept. For the graph properties computed within each ICA-identified network, we also included the “ICA-identified network” type as a fixed factor.

**Figure 2:**
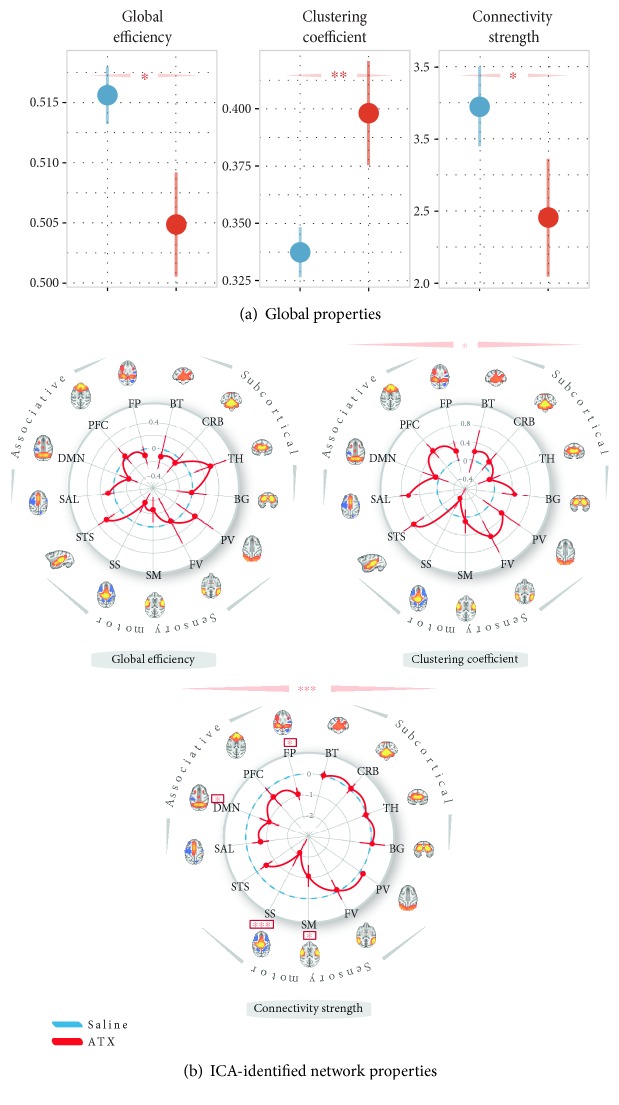
ATX effect on network architecture—(a) global graph properties. Global efficiency, clustering coefficient, and connectivity strength, under saline (blue) and ATX (red) pharmacological conditions. (b) ICA-identified network properties. The three spider plots represent the global efficiency, the clustering coefficient, and the connectivity strength computed for each ICA-identified resting-state networks (see Guedj et al. [49]). Importantly, these scores were expressed as a difference between the ATX condition and the saline control condition. Blue lines represent no difference between the two pharmacological conditions (difference equals to 0). Red stars indicate statistical differences between saline and ATX conditions: stars above the spider plots indicate a main effect of the pharmacological condition while stars above the networks indicated an interaction between the pharmacological condition and the ICA-identified network type (∗∗∗ = *p* value < 0.0001; ∗∗ = *p* value < 0.001; ∗ = *p* value < 0.05; • = *p* value < 0.1). Throughout this figure, the results are plotted as mean ± SEM. ATX = atomoxetine, BG = basal ganglia, BT = brainstem, CRB = cerebellum, DMN = default-mode network, FP = frontoparietal, FV = foveal visual, ICA = independent component analysis, PFC = prefrontal cortex, PV = peripheral visual, SAL = salience, SM = somatomotor, SS = somatosensory, STS = superior temporal sulcus, and TH = thalamus.

We found that boosting NE transmission altered the global brain topology, shifting its functional architecture toward a stronger local efficiency (Figure 2(a)), by significantly reducing the global efficiency, while increasing the clustering coefficient. Enhanced local efficiency following ATX injection was also found within specific RSNs previously characterized as independent networks (i.e., ICA-identified networks, see [49], Figure 2(b)). We also observed a decrease in connectivity strength at the whole-brain level and within sensory-motor and associative brain networks (Figures 2(a) and 2(b)) following ATX injection in accordance with our previous results [49]. As postulated by Eldar et al. (2013), the increase in the clustering coefficient could reflect an increase in neural gain. In other words, and together with our previous findings [49], we suggest that boosting NE transmission triggers large-scale brain network reorganizations, enhances the local neuronal communication at the whole-brain level, and adjusts functional connectivity within sensory-motor and associative brain networks. Importantly, this finding corroborates the idea that the LC-NE system plays a key role in shaping cortical states via its highly distributed projections throughout virtually all the brains [55, 56, 63, 68]. While our results are consistent with those of Eldar et al. [62], they contrast with a recent study that has also investigated the effect of ATX on the whole brain at rest [69]. Similar to our study, Van Den Brink et al. [69] compared the brain topology of healthy human subjects at rest using fMRI before and after the administration of a dose of ATX, in a similar range as that administered to our animals. They found that ATX led to a decrease of the clustering coefficient measured on region-level graphs using an atlas-based brain parcellation (90 regions). One possibility is that the discrepancy between the two studies is due to the difference in the definition of the graphical nodes. The clustering coefficient might indeed vary as a function of spatial scale [64]. In Van Den Brink et al.'s study, they used an atlas-based brain parcellation (90 regions) while in our study, we used a finer-grained spatial resolution (471 regions). Furthermore, all graph properties are calculated on a matrix where a threshold is traditionally applied to obtain a sparse network, therefore considering only the strongest brain connections. It is therefore also possible that this discrepancy simply reflects differences in graph densities. Future works should further investigate spatial effects of NE administration on functional connectivity depending on graph density and the choice of parcellation scale.

## 5. Correlations in Band-Limited Amplitude Envelope of the Gamma Rhythm: A Key Role in the NE-Dependent Local-to-Global Neuronal Dynamics?

**Figure 3:**
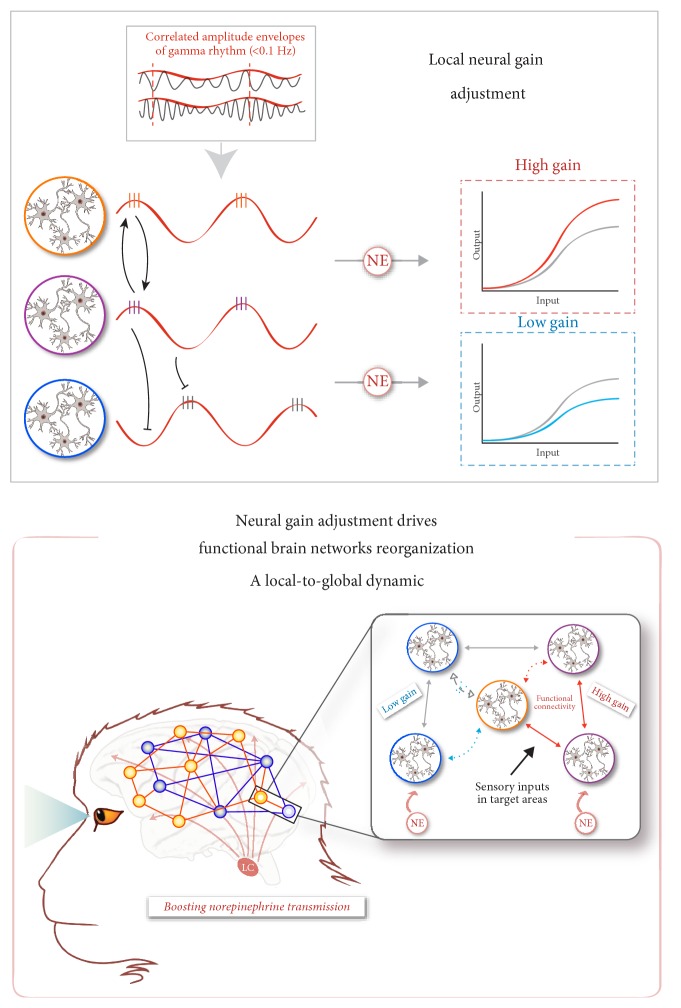
Mechanism proposed for the NE-dependent local-to-global neuronal dynamics—top panel—relationship between the amplitude envelope correlations in gamma rhythm and NE-dependent local neural gain adjustments. The orange and purple neuronal populations, whose amplitude envelopes cofluctuate, allow the increase in neural gain induced by the norepinephrine system activation to be effective and to spread (upper-right inset, red trace), whereas the purple and blue neuronal populations that exhibited a distinct temporal dynamic do not allow the increase in neural gain to be expressed locally, and this results in a decrease in neural gain (bottom-right inset, blue trace)—bottom panel—boosting norepinephrine transmission is thought to induce an increase in neural gain. The insert illustrates an example of the effect of neural gain adjustment on interactions between different neuronal populations. The five groups of neurons are anatomically interconnected. Sensory input signals influence the activity of target regions (purple and orange groups of neurons). Norepinephrine transmission also modulates the activity of groups of target neurons (violet and blue groups of neurons), increasing or decreasing the neural gain, respectively. The amplification of gain in the neuronal violet group then induces an increase in the functional connectivity between violet and orange groups (red arrows). Similarly, the reduction in neural gain in the blue neuronal group induces a decrease in functional connectivity between blue and orange groups (blue arrow and cross). Thus, under the influence of this local modulation of neural gain, neural networks reconfigure, creating a new organization of functional networks at the whole-brain scale.

Thus far, we found that on the one hand, boosting NE transmission led to large-scale brain network reorganizations, and on the other hand, it increased the local efficiency that could reflect an improvement of the neural gain. In the next sections, based on the assumption that the modulation of neural gain could represent the mechanism underlying the flexibility of neural networks [57–59], we propose that the two mechanisms are interdependent such that the increase of neural gain inferred from the clustering coefficient could induce large-scale brain network reorganizations, facilitating a wide range of cognitive processes (Figure 3). The demonstration of the co-occurrence of these two mechanisms following the stimulation of the LC-NE system is an important first step toward this assumption. It also provides a unified framework of the LC-NE theories [32, 33] and underlines a central feature of this system on the dynamics of the brain functional connectivity.

### 5.1. Spontaneous Brain Activity and Gamma Rhythm

It has been suggested that slow fluctuations in brain activity might be under brainstem control and may be related to behavioral variations [63, 70–72]. Here, we further suggest that a LC-NE-dependent adjustment of the neural gain could drive functional brain network reorganizations through coherence in the gamma rhythm (>30 Hz). Although the correspondence between the hemodynamic response measured using fMRI and the neuronal dynamic measured locally using electrophysiological recordings is far from clear, there exists some evidence suggesting correspondence between correlations in fMRI signals (i.e., functional connectivity) and correlations between the amplitude envelopes of band-limited cortical activity at distant points in the brain [70, 73, 74]. The correlations between the amplitude envelopes of band-limited cortical activity are a measure of the comodulation of the amplitude envelopes of oscillations in two areas, often spatially remote [75]. The covariations between the amplitude envelopes are very slow, within a similar range as those observed in resting-state fMRI fluctuations, with a frequency below 0.1 Hz [76]. At rest, electrophysiological studies in both humans and animals revealed that the amplitude envelopes in the gamma rhythm exhibit spatial coherence between functionally related areas [72, 74, 77, 78]. As RSNs, these fluctuations display consistent interhemispheric correlations and spatial specificities [74]. In particular, Schölvinck et al. [72] recently provided evidence of a more consistent relationship between spontaneous fMRI signals and gamma-range band-limited power by recording from multiple cortical areas in the awake monkey during “resting-state” fMRI scans using implanted electrode arrays. They also reported correlations, though less consistent, between spontaneous fMRI signals and the band-limited power derived from other frequency bands, which may suggest frequency division multiplexing [79], that would serve to convey information through separate frequency bands.

### 5.2. Could Neural Gain Adjustment Drive Functional Brain Network Reorganization?

Fries [57] proposed that the presence or absence of correlations in gamma-range band-limited power serves as a mechanism for the local neural gain adjustment within and between neuronal populations. Thus, a local increase in neural gain could influence more distal neuronal populations whose amplitude envelopes cofluctuate, whereas such impact would be less effective in neuronal populations whose amplitude envelopes fluctuate with a distinct temporal dynamic (Figure 3(a)). Interestingly, a recent optogenetic manipulation modulating the level of gamma rhythmic inputs suggests that gamma oscillations enhance signal transmission by increasing neural gain [80]. The gamma rhythm is mainly governed by inhibitory interneurons that generate synchronized activity by imposing rhythmic inhibition onto the entire local network. As a consequence, pyramidal cell responses can only occur during periods of fading inhibition [81]. These “windows of opportunity” play a critical role in shaping neuronal network dynamics [58]. A study demonstrated, in awake cats and monkeys, that short- and long-range neural interactions depend on the phase relation of pairs of recording sites in the visual cortex, such that effective connectivity is maximal for the phase relation at which the two sites typically synchronize [82]. The ubiquity of this oscillatory activity could facilitate a fine modulation of the neuronal responsiveness at the whole-brain scale via a balance between high- and low-gain levels to shape neuronal activity depending on the context [82–85]. Accordingly, as shown in Figure 3(b), we propose that the interplay between high- and low-neural gains driven by the amplitude correlations, associated with the spread of gamma-band synchronization, could fine tune the functional connectivity between the brain areas, therefore inducing the large-scale brain network reorganizations that we reported at rest under a NE challenge [49].

Apart from the relationship that might exist between the local adjustment of neural gain and the correlations in gamma-range band-limited power, Voloh and Womelsdorf [86] proposed a role for the “phase resetting” of oscillatory activities in the coordination of large-scale brain network. Phase resetting refers to the realignment of ongoing oscillatory activities in relation to a given event, and it is thought to facilitate the transmission of a combination of multiple signals through a common neural substrate over large anatomical distances [79]. Such a phenomenon has been demonstrated between the anterior cingulate cortex and the lateral prefrontal cortex of monkeys in a task involving covert stimulus selection [87]. As suggested by Voloh and Womelsdorf [86], these mechanisms might participate in reorganizing oscillatory activity across the brain depending on the context [86]. These mechanisms could also be under the influence of neuromodulators. In some way, this phase resetting could be related to the “reset signal” driven by the LC-NE system as proposed by Bouret and Sara [33].

In sum, the co-occurrence of NE-dependent changes in local and global neuronal resting-state dynamics suggests a functional relationship between these two mechanisms. Via the temporal dynamic of gamma-range band-limited power, the release of NE could adjust the neural gain, promoting interactions only within the neuronal populations whose amplitude envelopes are correlated, thus making it possible to reorganize neuronal ensembles, functional networks, and ultimately, behavioral responses. The co-occurrence of both the local and global changes in functional connectivity patterns that we described above following a NE challenge at rest fits with this hypothesis. They also leave open questions about how these mechanisms are recruited during goal-directed behavior and how they adjust in different task contexts. As reviewed above, depending on its activity (i.e., tonic and phasic modes), the LC has been associated with different levels of behavioral flexibility. Its properties also allow this system to act at multiple time scales [1, 32], thus inducing behavioral transitions between tasks or within a given task in response to relevant stimuli [33, 41, 88]. Accordingly, a modulation of the tonic LC activity could adjust the neural gain, inducing the reconfiguration of functional networks toward a brain state adapted to the current context (extratask transition), while phasic LC firing could fine tune an established functional circuit in order to modulate its activity within a shorter timescale in response to a relevant stimulus in the environment (intratask transition). It is likely that depending on the task context, the LC-NE system shapes these local-to-global neuronal dynamics at the whole-brain level and this local-to-global adjustment could involve different oscillatory bands and involve the interaction with other neuromodulators [89].

## 6. Conclusions: Functional Implications of the Role of Gamma Rhythm in the NE-Dependent Brain Mechanisms

While the impact of the LC-NE system on cognitive processes is far from clear, we proposed here a unified framework integrating the putative influence of the LC-NE system on both local- and long-range adjustments of brain dynamics. Local NE-dependent adjustment of the neural gain toward a more structured and effective neuronal communication could drive long-range reorganization of functional brain networks via the gamma rhythm amplitude envelopes. The NE-dependent flexibility in the RSN functional topology and interactions that we have highlighted could be governed by the dynamics of gamma rhythm oscillations which has often been proposed as a mechanism for assembling neurons into synchronous networks capable of conducting information throughout target regions [57, 74, 82]. To the best of our knowledge, NE-evoked modulation of gamma rhythm has not yet been demonstrated in the behaving state. However, we believe that there exists converging evidence making our framework plausible. On the one hand, NE-dependent modulation of oscillatory activity has been shown in different frequency bands. For instance, Bari and Aston-Jones [90] demonstrated modulation of the LC neurons firing rate and sensory-evoked LFPs, spike-field and EEG-field coherences in cortical regions of the rat following ATX injection. Brown et al. [91] demonstrated that the stimulation of the LC affected different rhythms in the hippocampus (*θ* rhythm and *β* and *γ* frequencies, and see also [92–94]). On the other hand, changes in oscillatory activity across different frequency bands have been repeatedly linked to changes in goal-directed behavior (e.g., [95–97]). In particular, gamma oscillations have been observed in a variety of processes, from sensory perception [98] to selective attention [99, 100], maintenance of working memory [81, 101, 102]. In the attentional domain, gamma-band synchronization among neurons is enhanced in the primate brain during tasks involving the selection of a target stimulus among distractors [97, 99, 103] and can mediate long-range communication across distant brain areas [97]. And finally, as reviewed by Başar and Güntekin [104], abnormalities in the oscillatory dynamics have been described in a variety of disorders including the attention deficit hyperactivity disorder that appears more closely linked to dysfunctions of the catecholaminergic system. Here, by assembling these evidences, we further suggest that neuromodulation might help fine tune the oscillatory dynamics. We believe that our integrated framework on the role of the LC-NE system on local- and long-range adjustments of brain dynamics posits a new interesting hypothesis that could be directly tested using multisite electrophysiological recordings combined with pharmacological manipulations.

## Supplementary Material

‘Materiel and Methods' section.
