# Supplementary material for: Could LC-NE-Dependent Adjustment of Neural Gain Drive Functional Brain Network Reorganization?
Source: Neural Plast. 2017 Apr 30;2017:4328015. doi: 10.1155/2017/4328015 (PMC5457760; doi:10.1155/2017/4328015)
Supplement: Supplementary file 1 — ‘Materiel and Methods' section. [file 4328015.f1.docx]

**Supplementary material**

**Materiel and Methods**

*Subjects and ethics*

Three female rhesus monkeys (*Macaca mulatta*) participated in the study: CE (age, 10 years; weight, 5.5 kg), CA (12 years; 5.5 kg), and CI (5 years, 6 kg). CE underwent a surgery under anesthesia and aseptic conditions during which a plastic head post was secured by plastic screws and bone cement on its skull. CA and CI were tested with a non-invasive head-restrained helmet system [1]. This study was conducted in strict accordance with Directive 2010/63/UE of the European Parliament and the Council of 22 September 2010 on the protection of animals used for scientific purposes. The project was authorized by the French Ministry for Higher Education and Research (project no. 20-12-0401-005), based on a ethical evaluation by the French Committee on the Ethics of Experiments in Animals (C2EA) CELYNE registered at the national level as C2EA number 42.

*Drug administration*

ATX (0.5 mg⁄kg, Tocris Bioscience, Ellisville, MO), an inhibitor of NE reuptake, or saline (control condition) injections was administered intramuscularly one hour before the scanning session. There was at least one-week washout period between saline or ATX injections. ATX was diluted in sterile saline immediately prior to injection. Monkeys received either 0.1 ml/kg ATX solution or 0.5 ml of saline.

*Scanning procedures*

Before each scanning session, an exogenous contrast agent (Feraheme®, 10 mg/kg) was injected into the saphenous vein to increase the contrast-to-noise ratio and optimize the localization of the fMRI signal. During the scans, the alert monkey was placed into the magnet bore in the dark. Imaging data were collected on a 1.5 T MAGNETOM scanner (Siemens AG, Erlangen, Germany). Functional data from the whole brain were acquired with a gradient echo sequence lasting 13.33 min and a custom-made 9-cm receive surface coil (Rapid Biomed) (TR = 2 s; TE = 27 ms; 2 × 2 × 3 mm; 400 TRs per run). These long-lasting runs ensure high test-retest reliability scores [2]. A total of 6 imaging runs were acquired over 2 or 3 different days for each monkey per condition (ATX and saline conditions). Monkeys were rewarded only in between runs.

*Preprocessing*

Data were preprocessed using the AFNI software [3]. The preprocessing pipeline included despiking, motion correction, and temporal filtering to extract the spontaneous slowly fluctuating brain activity (0.01–0.1 Hz). Data from all three monkeys were then realigned to the INIA 19 rhesus monkey template including tissue probability maps [4] http://nitrc.org/projects/inia19/). A linear regression was applied to remove nuisance variables (the six parameter estimates for head motion, and the cerebrospinal fluid and white matter signals from the INIA 19 segmentation).

*Graph construction*

One graph per monkey and per run was constructed with voxels of the gray matter tissue probability map of the INIA 19 rhesus monkey template. First, voxel-level matrices were constructed and were then resampled to areas of 4 × 4 × 6 mm^3^ volume (8 voxels) to minimize artifactual correlations between neighboring voxels [5] and reduce the computational costs, while retaining a fine granularity. Matrices of 471 areas, for each monkey and run, were obtained where regional mean time-series were estimated by averaging the signal over all voxels within each area.

To construct the graphs, we computed normalized correlation coefficient (fisher r-to-z transformation) between the regional mean time-series of each pair of areas. A threshold was then applied based on the absolute values of their correlation coefficient to retain only the 10% of the highest correlation scores. This density was selected as it was the smallest density that maximizes the number of connected areas [6] (see figure S1). This threshold allowed to minimize the number of spurious edges in each network, to compare networks between pharmacological conditions while maintaining an equivalent number of edges in each network [7]. These sparse matrices were then used to calculate graph properties. Thus, the graphs were weighted (the value of an edge is not binary but equal to the correlation between the peaks) and undirected (the correlation between two areas do not take into account the direction of the connection).

*Graph properties*

Graph properties were calculated using the Radatools software (version 3.2; http://deim.urv.cat/~sergio.gomez/radatools.php), Python libraries *NumPy*, *Nipype* [8] and *igraph*. Specifically, we estimated the following properties for each graph: the global efficiency, the clustering coefficient and the connectivity strength [9]–[11]. These properties were computed for the whole brain and for each ICA-identified networks. For each ICA-identified network, we computed the graph properties including nodes within each of the network previously identified with the Independent Component Analysis (ICA) approach [12]. We created a mask based on each ICA map using a threshold of p < 0.5 with an alternative-hypothesis testing approach. Only voxel with positive z-scores were included and isolated voxels were excluded (<1%). The graph properties were then computed for the nodes included within each mask of the corresponding ICA-identified network.

The global efficiency reflects the level of global integration in the network. It measures the network’s ability to transfer information between nodes via multiple series of edges. The global efficiency *Eglob(G)* corresponds to the averaged inverse shortest path length between all pairs of nodes in the network:

$$E_{glob}\left( G \right)= \frac{1}{N(N-1)}\sum_{i\epsilon G,j\epsilon G,i\neq j} \frac{1}{d_{\mathrm{ij}}} ;$$

with *d_ij_* is the length of the shortest path between node *i* and node *j*.

The clustering coefficient *Clust(G)* reflects the number of connections that exists between the nearest neighbors of a node as a proportion of the maximum number of possible connections (Bullmore and Sporns, 2009). It is considered as a measure of the local connectivity and as such provides information about the local efficiency of a network.

$$C\left( G \right)= \frac{1}{N}\sum_{i\epsilon G} C_{i} ; \mathrm{where} C_{i}=\frac{number of edges in G_{i}}{k_{i}(k_{i}-1)/2} ;$$

with *C_i_* is the clustering coefficient of a specific node *i*, *G_i_* is the subgraph of neighbors of the node *i*, and *k_i_* is the number of neighbors of *i.*

Finally, the connectivity strength *S_i_* of a specific node *i* is defined as the mean of the correlation coefficient between the node *i* and all the other nodes within the network:

$${S\left( G \right)= \frac{1}{N}\sum_{i\epsilon G} S_{i} ; \mathrm{where} S}_{i}= \frac{1}{N-1}\sum_{i\epsilon G,j\epsilon G,i\neq j} s_{\mathrm{ij}} ;$$

with *s_ij_* is the correlation coefficient of the edge linking the node *j* to *i*.

*Statistical analysis*

We examined the effect of ATX on the graph properties computed above using a linear mixed model. The model included the pharmacological condition as a fixed factor and the subject as a random intercept to take into account inter-individual differences. For the properties calculated within each ICA-identified network, the model also included the ‘ICA-identified network’ type as a fixed factor. Post-hoc comparisons were carried out using pairwise comparisons through the ‘lsmeans’ package for R (p-adjusted with false discovery rate method).

**References**

[1] F. Hadj-Bouziane *et al.*, “The helmet head restraint system: a viable solution for resting state fMRI in awake monkeys,” *NeuroImage*, vol. 86, pp. 536–543, Feb. 2014.

[2] R. M. Birn, J. B. Diamond, M. A. Smith, and P. A. Bandettini, “Separating respiratory-variation-related fluctuations from neuronal-activity-related fluctuations in fMRI,” *NeuroImage*, vol. 31, no. 4, pp. 1536–1548, Jul. 2006.

[3] R. W. Cox, “AFNI: software for analysis and visualization of functional magnetic resonance neuroimages,” *Comput. Biomed. Res. Int. J.*, vol. 29, no. 3, pp. 162–173, Jun. 1996.

[4] T. Rohlfing *et al.*, “The INIA19 Template and NeuroMaps Atlas for Primate Brain Image Parcellation and Spatial Normalization,” *Front. Neuroinformatics*, vol. 6, Dec. 2012.

[5] A. Zalesky *et al.*, “Whole-brain anatomical networks: does the choice of nodes matter?,” *NeuroImage*, vol. 50, no. 3, pp. 970–983, Apr. 2010.

[6] S. Achard, R. Salvador, B. Whitcher, J. Suckling, and E. Bullmore, “A Resilient, Low-Frequency, Small-World Human Brain Functional Network with Highly Connected Association Cortical Hubs,” *J. Neurosci.*, vol. 26, no. 1, pp. 63–72, Jan. 2006.

[7] S. Achard and E. Bullmore, “Efficiency and Cost of Economical Brain Functional Networks,” *PLoS Comput. Biol.*, vol. 3, no. 2, Feb. 2007.

[8] K. Gorgolewski *et al.*, “Nipype: a flexible, lightweight and extensible neuroimaging data processing framework in Python,” *Front. Neuroinformatics*, vol. 5, p. 13, 2011.

[9] D. J. Watts and S. H. Strogatz, “Collective dynamics of ‘small-world’ networks,” *Nature*, vol. 393, no. 6684, pp. 440–442, Jun. 1998.

[10] V. Latora and M. Marchiori, “Efficient behavior of small-world networks,” *Phys. Rev. Lett.*, vol. 87, no. 19, p. 198701, Nov. 2001.

[11] V. Latora and M. Marchiori, “Economic small-world behavior in weighted networks,” *Eur. Phys. J. B - Condens. Matter Complex Syst.*, vol. 32, no. 2, pp. 249–263, Mar. 2003.

[12] C. Guedj, E. Monfardini, A. J. Reynaud, A. Farnè, M. Meunier, and F. Hadj-Bouziane, “Boosting Norepinephrine Transmission Triggers Flexible Reconfiguration of Brain Networks at Rest,” *Cereb. Cortex*, Sep. 2016.
