# Supplementary figures and images for: Could LC-NE-Dependent Adjustment of Neural Gain Drive Functional Brain Network Reorganization?
Source: Neural Plast. 2017 Apr 30;2017:4328015. doi: 10.1155/2017/4328015 (PMC5457760; doi:10.1155/2017/4328015)

## Slide 1
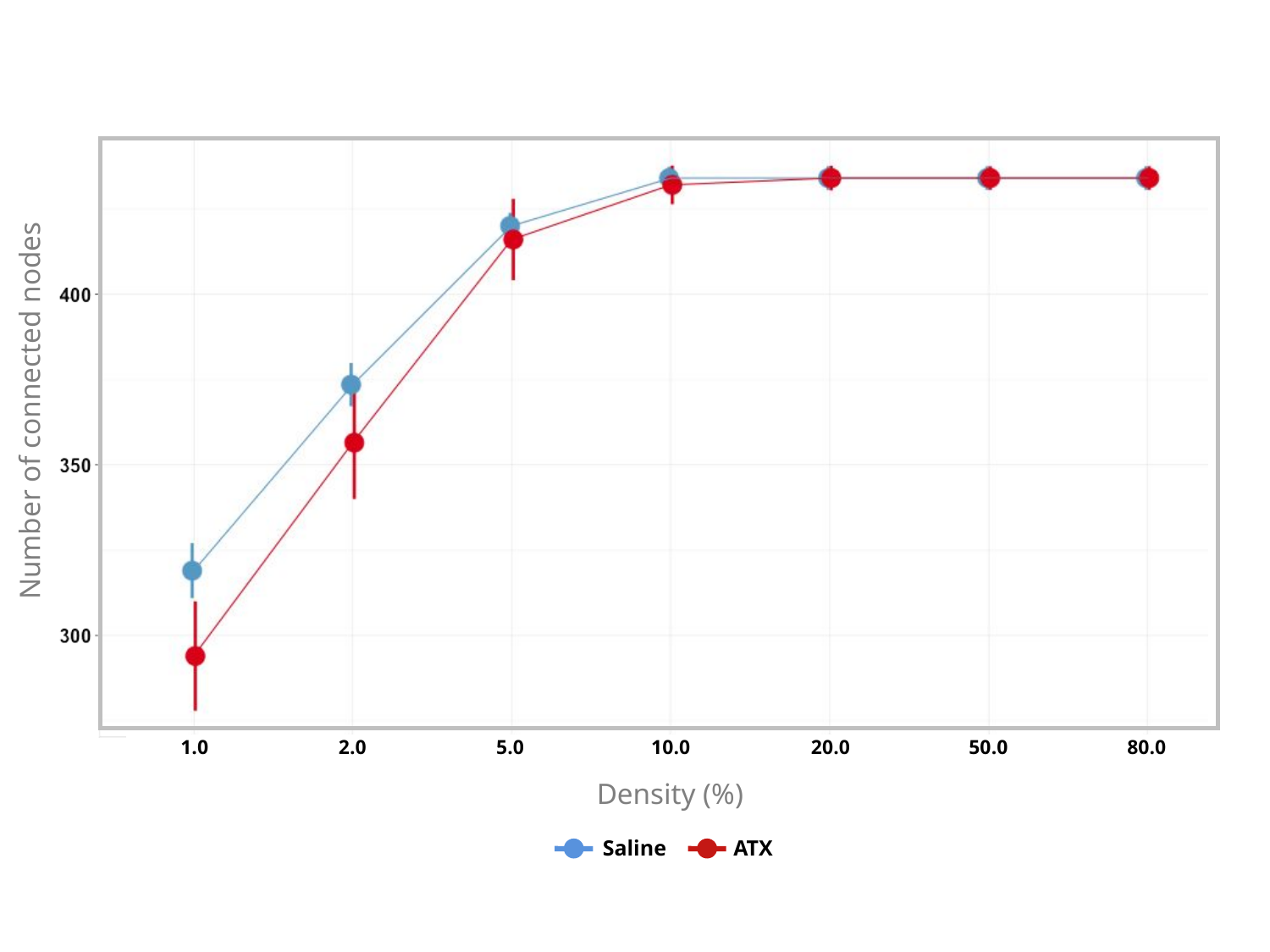

Number of connected nodes
1.0
2.0
5.0
10.0
20.0
50.0
80.0
Density (%)
Saline
ATX

Supplement: Supplementary file 2 [file 4328015.f2.ppt]

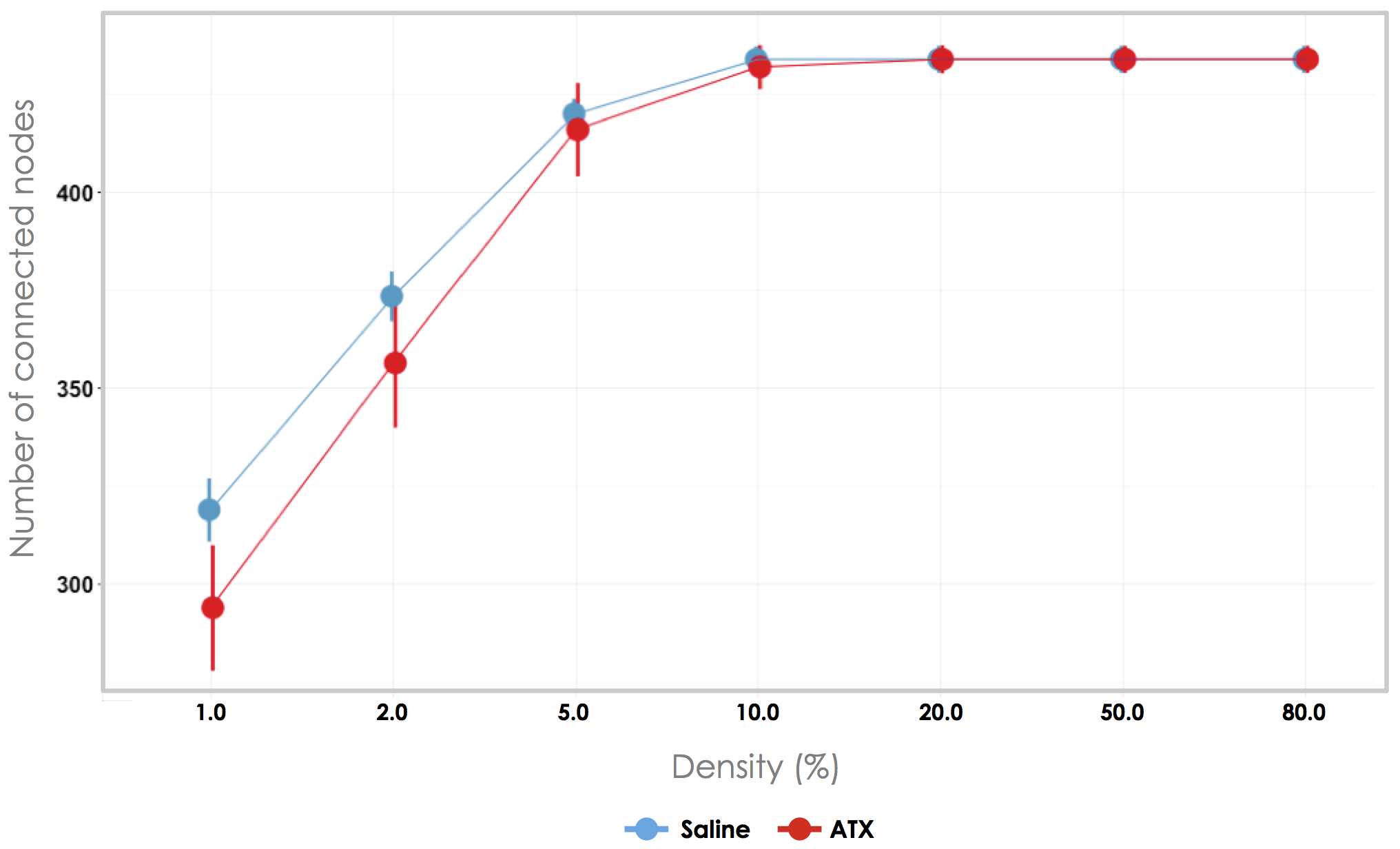

Supplement: Supplementary file 3 [file 4328015.f3.png]
